# 2-(3-Nitro­phen­yl)-4,5-diphenyl-1*H*-imidazol-3-ium chloride

**DOI:** 10.1107/S160053680803002X

**Published:** 2008-09-24

**Authors:** Li-Jing Cui

**Affiliations:** a Ordered Matter Science Research Center, College of Chemistry and Chemical, Engineering, Southeast University, Nanjing 210096, People’s Republic of China

## Abstract

The title compound, C_21_H_16_N_3_O_2_^+^·Cl^−^, contains two organic cations with similar conformation and two chloride ions in the asymmetric unit. The imidazole and benzene rings are twisted with respect to each other [dihedral angles of 24.05 (16), 24.31 (16) and 50.38 (13) in one cation and 27.70 (15), 25.07 (16) and 45.86 (14)° in the other]. The crystal packing is stabilized by N—H⋯Cl hydrogen bonds, forming an infinite one-dimensional chain parallel to the *c* axis.

## Related literature

For uses of imidazole derivatives, see: Dai & Fu (2008 ▶); Fu *et al.* (2008 ▶); Huang *et al.* (2008 ▶).
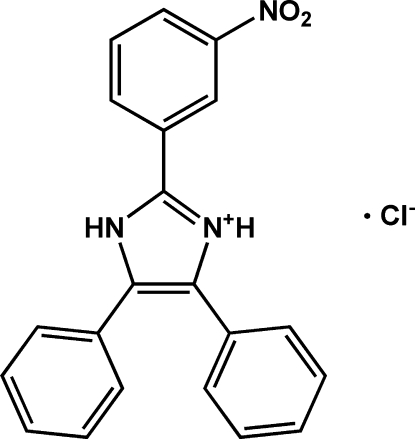


## Experimental

### 

#### Crystal data

C_21_H_16_N_3_O_2_^+^·Cl^−^
*M* *_r_* = 377.82
Triclinic, 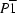

*a* = 9.7265 (19) Å
*b* = 12.364 (3) Å
*c* = 15.511 (3) Å
α = 91.97 (3)°
β = 90.15 (3)°
γ = 95.84 (3)°
*V* = 1854.5 (7) Å^3^
*Z* = 4
Mo *K*α radiation
μ = 0.23 mm^−1^
*T* = 298 (2) K
0.24 × 0.20 × 0.18 mm

#### Data collection

Rigaku Mercury2 diffractometer
Absorption correction: multi-scan (*CrystalClear*, Rigaku, 2005 ▶) *T*_min_ = 0.939, *T*_max_ = 0.957
18693 measured reflections
8132 independent reflections
5351 reflections with *I* > 2σ(*I*)
*R*_int_ = 0.043

#### Refinement

*R*[*F* ^2^ > 2σ(*F* ^2^)] = 0.062
*wR*(*F* ^2^) = 0.153
*S* = 1.07
8132 reflections
487 parameters
H-atom parameters constrained
Δρ_max_ = 0.18 e Å^−3^
Δρ_min_ = −0.27 e Å^−3^

### 

Data collection: *CrystalClear* (Rigaku, 2005 ▶); cell refinement: *CrystalClear*; data reduction: *CrystalClear*; program(s) used to solve structure: *SHELXS97* (Sheldrick, 2008 ▶); program(s) used to refine structure: *SHELXL97* (Sheldrick, 2008 ▶); molecular graphics: *ORTEP-3 for Windows* (Farrugia, 1997 ▶) and *SHELXTL* (Sheldrick, 2008 ▶); software used to prepare material for publication: *SHELXL97*.

## Supplementary Material

Crystal structure: contains datablocks I, global. DOI: 10.1107/S160053680803002X/dn2374sup1.cif

Structure factors: contains datablocks I. DOI: 10.1107/S160053680803002X/dn2374Isup2.hkl

Additional supplementary materials:  crystallographic information; 3D view; checkCIF report

## Figures and Tables

**Table 1:** Hydrogen-bond geometry (Å, °)

*D*—H⋯*A*	*D*—H	H⋯*A*	*D*⋯*A*	*D*—H⋯*A*
N3—H3⋯Cl1	0.86	2.30	3.066 (2)	148
N5—H5*A*⋯Cl1	0.86	2.20	3.045 (2)	166
N6—H6⋯Cl2	0.86	2.31	3.061 (2)	146
N2—H2⋯Cl2^i^	0.86	2.21	3.048 (2)	163

Symmetry code: (i) 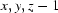.

**Fig. 1.**
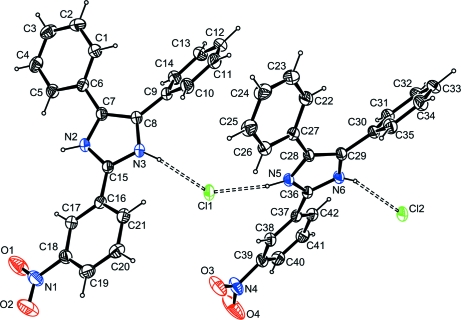
A view of the title compound with the atom-labeling scheme. Displacement ellipsoids are drawn at the 30% probability level. H atoms are represented as small sphere of arbitrary radii. Hydrogen bonds are shown as dashed line.

**Fig. 2.**
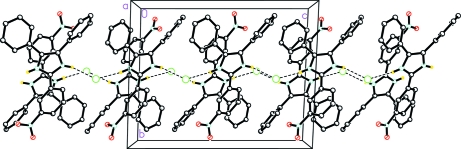
The crystal packing of the title compound viewed along the a axis showing the one-dimensionnal hydrogen bonding chain. Hydrogen atoms not involved in hydrogen bonding (dashed lines) have been omitted for clarity.

## supplementary crystallographic information

### Comment

Imidazole derivatives have found wide range of applications in coordination chemistry because of their multiple coordination modes as ligands to metal ions and for the construction of novel metal-organic frameworks (Huang, *et al.* 2008; Fu, *et al.* 2008; Dai & Fu 2008). We report here the crystal structure of the title compound, di-2-(3'-nitrophenyl)-4,5-diphenyl-1*H*-imidazole-3-ium dichloride.

The title compound contains two organic cation with similar conformation and two Cl^-^ ions in the asymmetric unit (Fig. 1). In both molecules the N atom of the imidazole is protonated. The imidazole and the benzene rings are twisted from each other by a dihedral angle of 24.05 (0.16) °, 24.31 (0.16) ° and 50.38 (0.13) ° (27.70 (0.15) °, 25.07 (0.16) ° and 45.86 (0.14) °) . The crystal packing is stabilized by N—H···Cl hydrogen bonds to form an infinite one-dimensional chain parallel to the *c* axis. (Table 1, Fig. 2).

### Experimental

Under nitrogen protection, benzil (20 mmol), 3-nitrobenzaldehyde (20 mmol) and amine acetate (50 mmol) were added in a flask. The mixture was stirred at 110 °C for 20 h in the solution of HAC (60 ml). The resulting solution was poured into ice water (200 ml), white solid was obtained after adding NaOH (6 mol/*L*) till PH=7, then filtered and washed with distilled water. The crude product was recrystallized with the solution of ethanol (150 ml) and hydrochloric acid (5 ml) to yield colorless block-like crystals, suitable for X-ray analysis.

### Refinement

All H atoms attached to C and N atoms were fixed geometrically and treated as riding with C–H = 0.93 Å (aromatic) and N–H = 0.86 Å with *U*_iso_(H) =1.2Ueq(C or N).

### Crystal data

**Table:**

C_21_H_16_N_3_O_2_^+^·Cl^−^	*Z* = 4
*M**_r_* = 377.82	*F*(000) = 784
Triclinic, *P*1	*D*_x_ = 1.353 Mg m^−^^3^
Hall symbol: -P 1	Mo *K*α radiation, λ = 0.71073 Å
*a* = 9.7265 (19) Å	Cell parameters from 3688 reflections
*b* = 12.364 (3) Å	θ = 2.5–27.1°
*c* = 15.511 (3) Å	µ = 0.23 mm^−^^1^
α = 91.97 (3)°	*T* = 298 K
β = 90.15 (3)°	Block, colorless
γ = 95.84 (3)°	0.24 × 0.20 × 0.18 mm
*V* = 1854.5 (7) Å^3^	

### Data collection

**Table:**

Rigaku Mercury2 diffractometer	8132 independent reflections
Radiation source: fine-focus sealed tube	5351 reflections with *I* > 2σ(*I*)
graphite	*R*_int_ = 0.043
Detector resolution: 13.6612 pixels mm^-1^	θ_max_ = 27.2°, θ_min_ = 2.5°
ω scans	*h* = −12→12
Absorption correction: multi-scan (CrystalClear, Rigaku, 2005)	*k* = −15→15
*T*_min_ = 0.939, *T*_max_ = 0.957	*l* = −19→19
18693 measured reflections	

### Refinement

**Table:**

Refinement on *F*^2^	Primary atom site location: structure-invariant direct methods
Least-squares matrix: full	Secondary atom site location: difference Fourier map
*R*[*F*^2^ > 2σ(*F*^2^)] = 0.062	Hydrogen site location: inferred from neighbouring sites
*wR*(*F*^2^) = 0.153	H-atom parameters constrained
*S* = 1.07	*w* = 1/[σ^2^(*F*_o_^2^) + (0.0593*P*)^2^ + 0.2499*P*] where *P* = (*F*_o_^2^ + 2*F*_c_^2^)/3
8132 reflections	(Δ/σ)_max_ = 0.009
487 parameters	Δρ_max_ = 0.18 e Å^−^^3^
0 restraints	Δρ_min_ = −0.27 e Å^−^^3^

### Special details

**Table:**

Geometry. All esds (except the esd in the dihedral angle between two l.s. planes) are estimated using the full covariance matrix. The cell esds are taken into account individually in the estimation of esds in distances, angles and torsion angles; correlations between esds in cell parameters are only used when they are defined by crystal symmetry. An approximate (isotropic) treatment of cell esds is used for estimating esds involving l.s. planes.
Refinement. Refinement of *F*^2^ against ALL reflections. The weighted *R*-factor *wR* and goodness of fit *S* are based on *F*^2^, conventional *R*-factors *R* are based on *F*, with *F* set to zero for negative *F*^2^. The threshold expression of *F*^2^ > σ(*F*^2^) is used only for calculating *R*-factors(gt) *etc*. and is not relevant to the choice of reflections for refinement. *R*-factors based on *F*^2^ are statistically about twice as large as those based on *F*, and *R*- factors based on ALL data will be even larger.

### Fractional atomic coordinates and isotropic or equivalent isotropic displacement parameters (Å^2^)

**Table:**

	*x*	*y*	*z*	*U*_iso_*/*U*_eq_	
Cl1	0.79840 (9)	0.52921 (6)	0.29960 (4)	0.0600 (2)	
N1	0.9375 (3)	0.8492 (2)	−0.07795 (17)	0.0613 (7)	
N2	0.6852 (2)	0.45441 (16)	−0.01555 (12)	0.0391 (5)	
H2	0.6765	0.4807	−0.0656	0.047*	
N3	0.7522 (2)	0.43586 (16)	0.11489 (12)	0.0403 (5)	
H3	0.7947	0.4480	0.1635	0.048*	
O1	0.8327 (3)	0.8432 (2)	−0.12130 (19)	0.1011 (10)	
O2	1.0299 (3)	0.9231 (2)	−0.08381 (19)	0.0942 (9)	
C1	0.4870 (3)	0.1792 (2)	−0.02959 (18)	0.0551 (7)	
H1	0.5298	0.1484	0.0159	0.066*	
C2	0.3941 (4)	0.1151 (3)	−0.0821 (2)	0.0669 (9)	
H2A	0.3744	0.0417	−0.0711	0.080*	
C3	0.3307 (3)	0.1583 (3)	−0.1503 (2)	0.0639 (8)	
H3A	0.2676	0.1149	−0.1849	0.077*	
C4	0.3613 (3)	0.2660 (3)	−0.16665 (18)	0.0561 (7)	
H4	0.3199	0.2952	−0.2134	0.067*	
C5	0.4534 (3)	0.3323 (2)	−0.11451 (16)	0.0454 (6)	
H5	0.4728	0.4055	−0.1265	0.055*	
C6	0.5168 (3)	0.2898 (2)	−0.04434 (15)	0.0408 (6)	
C7	0.6123 (2)	0.3593 (2)	0.01275 (15)	0.0391 (6)	
C8	0.6553 (3)	0.3488 (2)	0.09655 (15)	0.0387 (5)	
C9	0.6232 (3)	0.2638 (2)	0.15934 (15)	0.0401 (6)	
C10	0.7300 (3)	0.2159 (2)	0.19771 (17)	0.0506 (7)	
H10	0.8213	0.2418	0.1871	0.061*	
C11	0.7012 (4)	0.1304 (3)	0.25142 (19)	0.0624 (8)	
H11	0.7732	0.0985	0.2768	0.075*	
C12	0.5673 (4)	0.0918 (2)	0.26774 (19)	0.0620 (8)	
H12	0.5483	0.0330	0.3031	0.074*	
C13	0.4609 (3)	0.1404 (2)	0.23154 (19)	0.0594 (8)	
H13	0.3699	0.1147	0.2432	0.071*	
C14	0.4877 (3)	0.2260 (2)	0.17870 (17)	0.0505 (7)	
H14	0.4149	0.2591	0.1556	0.061*	
C15	0.7710 (2)	0.49879 (19)	0.04687 (14)	0.0368 (5)	
C16	0.8719 (3)	0.5943 (2)	0.04194 (15)	0.0381 (5)	
C17	0.8556 (3)	0.6750 (2)	−0.01615 (15)	0.0413 (6)	
H17	0.7795	0.6703	−0.0530	0.050*	
C18	0.9557 (3)	0.7625 (2)	−0.01757 (16)	0.0444 (6)	
C19	1.0704 (3)	0.7728 (2)	0.03556 (19)	0.0531 (7)	
H19	1.1362	0.8326	0.0325	0.064*	
C20	1.0856 (3)	0.6926 (2)	0.09320 (18)	0.0515 (7)	
H20	1.1621	0.6982	0.1298	0.062*	
C21	0.9871 (3)	0.6035 (2)	0.09695 (16)	0.0446 (6)	
H21	0.9977	0.5496	0.1362	0.054*	
Cl2	0.71004 (9)	0.52695 (6)	0.79879 (4)	0.0598 (2)	
N4	0.6666 (3)	0.8561 (2)	0.43176 (17)	0.0660 (7)	
N5	0.7989 (2)	0.45279 (15)	0.48411 (12)	0.0364 (5)	
H5A	0.8139	0.4793	0.4341	0.044*	
N6	0.7275 (2)	0.43327 (16)	0.61432 (12)	0.0392 (5)	
H6	0.6880	0.4449	0.6628	0.047*	
O3	0.7518 (3)	0.8431 (2)	0.37747 (17)	0.0901 (8)	
O4	0.6168 (4)	0.9416 (2)	0.4429 (2)	0.1358 (14)	
C22	0.9164 (3)	0.1767 (2)	0.46440 (17)	0.0496 (7)	
H22	0.8618	0.1449	0.5076	0.059*	
C23	0.9902 (3)	0.1127 (2)	0.41130 (19)	0.0600 (8)	
H23	0.9861	0.0382	0.4194	0.072*	
C24	1.0702 (3)	0.1585 (2)	0.3462 (2)	0.0590 (8)	
H24	1.1212	0.1153	0.3111	0.071*	
C25	1.0744 (3)	0.2681 (2)	0.33328 (18)	0.0547 (7)	
H25	1.1264	0.2986	0.2884	0.066*	
C26	1.0017 (3)	0.3328 (2)	0.38663 (16)	0.0436 (6)	
H26	1.0054	0.4070	0.3775	0.052*	
C27	0.9225 (2)	0.2886 (2)	0.45424 (15)	0.0379 (5)	
C28	0.8466 (2)	0.35808 (19)	0.51171 (14)	0.0364 (5)	
C29	0.8018 (2)	0.34605 (19)	0.59540 (15)	0.0369 (5)	
C30	0.8123 (3)	0.26139 (19)	0.65856 (15)	0.0383 (5)	
C31	0.6949 (3)	0.2205 (2)	0.70209 (16)	0.0466 (6)	
H31	0.6115	0.2491	0.6928	0.056*	
C32	0.7009 (3)	0.1376 (2)	0.75902 (18)	0.0568 (8)	
H32	0.6218	0.1112	0.7883	0.068*	
C33	0.8234 (4)	0.0940 (2)	0.77268 (18)	0.0594 (8)	
H33	0.8268	0.0369	0.8099	0.071*	
C34	0.9410 (3)	0.1354 (2)	0.73084 (18)	0.0571 (7)	
H34	1.0241	0.1064	0.7404	0.069*	
C35	0.9371 (3)	0.2198 (2)	0.67470 (17)	0.0482 (6)	
H35	1.0175	0.2484	0.6479	0.058*	
C36	0.7259 (2)	0.49708 (19)	0.54660 (14)	0.0363 (5)	
C37	0.6520 (2)	0.59366 (19)	0.54290 (14)	0.0366 (5)	
C38	0.6928 (3)	0.6761 (2)	0.48667 (15)	0.0407 (6)	
H38	0.7668	0.6706	0.4495	0.049*	
C39	0.6200 (3)	0.7663 (2)	0.48787 (17)	0.0459 (6)	
C40	0.5077 (3)	0.7769 (2)	0.54107 (18)	0.0517 (7)	
H40	0.4603	0.8385	0.5399	0.062*	
C41	0.4678 (3)	0.6940 (2)	0.59584 (17)	0.0496 (7)	
H41	0.3924	0.6993	0.6320	0.060*	
C42	0.5394 (3)	0.6034 (2)	0.59706 (16)	0.0438 (6)	
H42	0.5122	0.5481	0.6344	0.053*	

### Atomic displacement parameters (Å^2^)

**Table:**

	*U*^11^	*U*^22^	*U*^33^	*U*^12^	*U*^13^	*U*^23^
Cl1	0.0979 (6)	0.0547 (4)	0.0285 (3)	0.0122 (4)	−0.0013 (3)	0.0057 (3)
N1	0.0720 (17)	0.0502 (15)	0.0619 (16)	0.0012 (13)	0.0075 (14)	0.0166 (13)
N2	0.0499 (12)	0.0419 (12)	0.0257 (9)	0.0048 (9)	0.0000 (9)	0.0048 (9)
N3	0.0497 (12)	0.0445 (12)	0.0266 (10)	0.0028 (10)	−0.0027 (9)	0.0039 (9)
O1	0.0982 (19)	0.090 (2)	0.113 (2)	−0.0149 (15)	−0.0373 (17)	0.0584 (18)
O2	0.0948 (18)	0.0660 (16)	0.119 (2)	−0.0184 (14)	−0.0024 (16)	0.0381 (16)
C1	0.075 (2)	0.0441 (16)	0.0457 (15)	0.0051 (14)	−0.0094 (14)	0.0028 (13)
C2	0.090 (2)	0.0437 (17)	0.065 (2)	−0.0026 (16)	−0.0108 (18)	−0.0046 (15)
C3	0.0647 (19)	0.061 (2)	0.064 (2)	0.0034 (16)	−0.0181 (16)	−0.0159 (16)
C4	0.0605 (18)	0.066 (2)	0.0437 (15)	0.0183 (15)	−0.0123 (13)	−0.0069 (14)
C5	0.0487 (15)	0.0482 (15)	0.0408 (14)	0.0120 (12)	−0.0002 (12)	0.0018 (12)
C6	0.0451 (14)	0.0449 (14)	0.0328 (12)	0.0078 (11)	0.0000 (11)	−0.0025 (11)
C7	0.0450 (14)	0.0411 (14)	0.0321 (12)	0.0082 (11)	0.0004 (10)	0.0038 (11)
C8	0.0459 (14)	0.0388 (13)	0.0315 (12)	0.0031 (11)	−0.0017 (10)	0.0046 (10)
C9	0.0527 (15)	0.0376 (13)	0.0303 (12)	0.0049 (11)	−0.0011 (11)	0.0028 (10)
C10	0.0568 (17)	0.0524 (16)	0.0440 (15)	0.0107 (13)	−0.0031 (13)	0.0079 (13)
C11	0.090 (2)	0.0538 (18)	0.0466 (16)	0.0225 (17)	−0.0152 (16)	0.0046 (14)
C12	0.102 (3)	0.0368 (15)	0.0455 (16)	−0.0027 (16)	−0.0046 (17)	0.0093 (13)
C13	0.072 (2)	0.0488 (17)	0.0537 (17)	−0.0120 (15)	0.0016 (15)	0.0090 (14)
C14	0.0564 (17)	0.0485 (16)	0.0466 (15)	0.0026 (13)	−0.0036 (13)	0.0096 (13)
C15	0.0450 (14)	0.0369 (13)	0.0293 (11)	0.0069 (10)	0.0005 (10)	0.0022 (10)
C16	0.0450 (14)	0.0386 (13)	0.0307 (12)	0.0053 (11)	0.0051 (10)	−0.0016 (10)
C17	0.0450 (14)	0.0453 (15)	0.0339 (12)	0.0053 (11)	0.0011 (11)	0.0032 (11)
C18	0.0542 (15)	0.0396 (14)	0.0396 (14)	0.0051 (12)	0.0043 (12)	0.0039 (11)
C19	0.0544 (17)	0.0483 (16)	0.0545 (17)	−0.0027 (13)	0.0009 (13)	−0.0054 (14)
C20	0.0520 (16)	0.0506 (17)	0.0509 (16)	0.0033 (13)	−0.0091 (13)	−0.0058 (14)
C21	0.0524 (15)	0.0469 (15)	0.0358 (13)	0.0117 (12)	−0.0025 (11)	−0.0021 (11)
Cl2	0.0970 (6)	0.0540 (4)	0.0279 (3)	0.0041 (4)	0.0030 (3)	0.0048 (3)
N4	0.087 (2)	0.0554 (16)	0.0615 (16)	0.0275 (14)	0.0091 (15)	0.0225 (13)
N5	0.0458 (11)	0.0370 (11)	0.0270 (10)	0.0059 (9)	0.0019 (8)	0.0045 (8)
N6	0.0505 (12)	0.0411 (11)	0.0270 (10)	0.0079 (9)	0.0070 (9)	0.0043 (9)
O3	0.119 (2)	0.0720 (16)	0.0863 (17)	0.0297 (15)	0.0429 (16)	0.0379 (14)
O4	0.194 (3)	0.0807 (19)	0.151 (3)	0.082 (2)	0.083 (3)	0.066 (2)
C22	0.0675 (18)	0.0410 (15)	0.0408 (14)	0.0077 (13)	0.0075 (13)	0.0028 (12)
C23	0.083 (2)	0.0437 (16)	0.0544 (18)	0.0159 (15)	0.0040 (16)	−0.0031 (14)
C24	0.0666 (19)	0.0547 (18)	0.0568 (18)	0.0163 (15)	0.0124 (15)	−0.0132 (15)
C25	0.0547 (17)	0.0615 (18)	0.0471 (16)	0.0035 (14)	0.0128 (13)	−0.0052 (14)
C26	0.0478 (15)	0.0417 (14)	0.0413 (14)	0.0038 (11)	0.0016 (11)	0.0026 (11)
C27	0.0429 (13)	0.0379 (13)	0.0330 (12)	0.0046 (10)	−0.0013 (10)	0.0003 (10)
C28	0.0443 (14)	0.0357 (13)	0.0296 (11)	0.0041 (10)	0.0003 (10)	0.0053 (10)
C29	0.0444 (13)	0.0365 (13)	0.0302 (11)	0.0060 (10)	0.0010 (10)	0.0039 (10)
C30	0.0503 (14)	0.0334 (13)	0.0313 (12)	0.0047 (11)	0.0016 (10)	0.0040 (10)
C31	0.0538 (16)	0.0450 (15)	0.0407 (14)	0.0016 (12)	0.0041 (12)	0.0050 (12)
C32	0.076 (2)	0.0440 (16)	0.0474 (16)	−0.0111 (15)	0.0093 (15)	0.0098 (13)
C33	0.099 (2)	0.0337 (14)	0.0455 (16)	0.0044 (15)	−0.0031 (16)	0.0085 (12)
C34	0.076 (2)	0.0471 (17)	0.0515 (17)	0.0191 (15)	−0.0077 (15)	0.0054 (14)
C35	0.0551 (16)	0.0481 (15)	0.0427 (14)	0.0093 (13)	0.0043 (12)	0.0061 (12)
C36	0.0444 (14)	0.0376 (13)	0.0272 (11)	0.0049 (10)	0.0003 (10)	0.0026 (10)
C37	0.0429 (13)	0.0379 (13)	0.0294 (11)	0.0071 (10)	−0.0020 (10)	−0.0011 (10)
C38	0.0452 (14)	0.0441 (14)	0.0340 (12)	0.0103 (11)	0.0020 (11)	0.0039 (11)
C39	0.0572 (16)	0.0428 (15)	0.0396 (14)	0.0126 (12)	−0.0023 (12)	0.0070 (12)
C40	0.0575 (17)	0.0493 (16)	0.0513 (16)	0.0215 (13)	−0.0036 (13)	−0.0022 (13)
C41	0.0485 (15)	0.0551 (17)	0.0465 (15)	0.0129 (13)	0.0055 (12)	−0.0042 (13)
C42	0.0486 (15)	0.0445 (15)	0.0373 (13)	0.0012 (12)	0.0014 (11)	−0.0002 (11)

### Geometric parameters (Å, °)

**Table:**

N1—O1	1.214 (3)	N4—O3	1.201 (3)
N1—O2	1.221 (3)	N4—O4	1.215 (3)
N1—C18	1.471 (3)	N4—C39	1.470 (3)
N2—C15	1.342 (3)	N5—C36	1.337 (3)
N2—C7	1.395 (3)	N5—C28	1.384 (3)
N2—H2	0.8600	N5—H5A	0.8600
N3—C15	1.334 (3)	N6—C36	1.336 (3)
N3—C8	1.378 (3)	N6—C29	1.381 (3)
N3—H3	0.8600	N6—H6	0.8600
C1—C2	1.384 (4)	C22—C23	1.376 (4)
C1—C6	1.395 (4)	C22—C27	1.393 (3)
C1—H1	0.9300	C22—H22	0.9300
C2—C3	1.374 (4)	C23—C24	1.378 (4)
C2—H2A	0.9300	C23—H23	0.9300
C3—C4	1.366 (4)	C24—C25	1.373 (4)
C3—H3A	0.9300	C24—H24	0.9300
C4—C5	1.389 (4)	C25—C26	1.378 (4)
C4—H4	0.9300	C25—H25	0.9300
C5—C6	1.394 (3)	C26—C27	1.396 (3)
C5—H5	0.9300	C26—H26	0.9300
C6—C7	1.472 (3)	C27—C28	1.468 (3)
C7—C8	1.379 (3)	C28—C29	1.379 (3)
C8—C9	1.468 (3)	C29—C30	1.469 (3)
C9—C10	1.390 (4)	C30—C31	1.388 (3)
C9—C14	1.390 (4)	C30—C35	1.392 (4)
C10—C11	1.376 (4)	C31—C32	1.381 (4)
C10—H10	0.9300	C31—H31	0.9300
C11—C12	1.368 (4)	C32—C33	1.376 (4)
C11—H11	0.9300	C32—H32	0.9300
C12—C13	1.376 (4)	C33—C34	1.379 (4)
C12—H12	0.9300	C33—H33	0.9300
C13—C14	1.366 (4)	C34—C35	1.385 (4)
C13—H13	0.9300	C34—H34	0.9300
C14—H14	0.9300	C35—H35	0.9300
C15—C16	1.461 (3)	C36—C37	1.458 (3)
C16—C17	1.388 (3)	C37—C38	1.392 (3)
C16—C21	1.400 (3)	C37—C42	1.395 (3)
C17—C18	1.381 (4)	C38—C39	1.380 (3)
C17—H17	0.9300	C38—H38	0.9300
C18—C19	1.378 (4)	C39—C40	1.385 (4)
C19—C20	1.376 (4)	C40—C41	1.379 (4)
C19—H19	0.9300	C40—H40	0.9300
C20—C21	1.387 (4)	C41—C42	1.379 (4)
C20—H20	0.9300	C41—H41	0.9300
C21—H21	0.9300	C42—H42	0.9300
			
O1—N1—O2	122.6 (3)	O3—N4—O4	122.5 (3)
O1—N1—C18	118.8 (2)	O3—N4—C39	119.7 (2)
O2—N1—C18	118.6 (3)	O4—N4—C39	117.8 (3)
C15—N2—C7	110.03 (19)	C36—N5—C28	110.09 (19)
C15—N2—H2	125.0	C36—N5—H5A	125.0
C7—N2—H2	125.0	C28—N5—H5A	125.0
C15—N3—C8	110.7 (2)	C36—N6—C29	110.38 (19)
C15—N3—H3	124.7	C36—N6—H6	124.8
C8—N3—H3	124.7	C29—N6—H6	124.8
C2—C1—C6	120.5 (3)	C23—C22—C27	120.9 (3)
C2—C1—H1	119.8	C23—C22—H22	119.5
C6—C1—H1	119.8	C27—C22—H22	119.5
C3—C2—C1	120.9 (3)	C22—C23—C24	120.2 (3)
C3—C2—H2A	119.6	C22—C23—H23	119.9
C1—C2—H2A	119.6	C24—C23—H23	119.9
C4—C3—C2	119.3 (3)	C25—C24—C23	119.9 (3)
C4—C3—H3A	120.4	C25—C24—H24	120.0
C2—C3—H3A	120.4	C23—C24—H24	120.0
C3—C4—C5	121.0 (3)	C24—C25—C26	120.2 (3)
C3—C4—H4	119.5	C24—C25—H25	119.9
C5—C4—H4	119.5	C26—C25—H25	119.9
C4—C5—C6	120.3 (3)	C25—C26—C27	120.9 (3)
C4—C5—H5	119.8	C25—C26—H26	119.5
C6—C5—H5	119.8	C27—C26—H26	119.5
C5—C6—C1	118.1 (2)	C22—C27—C26	117.8 (2)
C5—C6—C7	121.1 (2)	C22—C27—C28	121.4 (2)
C1—C6—C7	120.8 (2)	C26—C27—C28	120.8 (2)
C8—C7—N2	105.8 (2)	C29—C28—N5	106.3 (2)
C8—C7—C6	132.3 (2)	C29—C28—C27	131.8 (2)
N2—C7—C6	121.9 (2)	N5—C28—C27	121.8 (2)
N3—C8—C7	106.5 (2)	C28—C29—N6	106.1 (2)
N3—C8—C9	121.0 (2)	C28—C29—C30	133.4 (2)
C7—C8—C9	132.4 (2)	N6—C29—C30	120.4 (2)
C10—C9—C14	118.7 (2)	C31—C30—C35	119.1 (2)
C10—C9—C8	119.6 (2)	C31—C30—C29	119.5 (2)
C14—C9—C8	121.6 (2)	C35—C30—C29	121.4 (2)
C11—C10—C9	120.2 (3)	C32—C31—C30	120.5 (3)
C11—C10—H10	119.9	C32—C31—H31	119.7
C9—C10—H10	119.9	C30—C31—H31	119.7
C12—C11—C10	120.4 (3)	C33—C32—C31	120.3 (3)
C12—C11—H11	119.8	C33—C32—H32	119.8
C10—C11—H11	119.8	C31—C32—H32	119.8
C11—C12—C13	119.7 (3)	C32—C33—C34	119.5 (3)
C11—C12—H12	120.1	C32—C33—H33	120.2
C13—C12—H12	120.1	C34—C33—H33	120.2
C14—C13—C12	120.6 (3)	C33—C34—C35	120.8 (3)
C14—C13—H13	119.7	C33—C34—H34	119.6
C12—C13—H13	119.7	C35—C34—H34	119.6
C13—C14—C9	120.3 (3)	C34—C35—C30	119.7 (3)
C13—C14—H14	119.9	C34—C35—H35	120.2
C9—C14—H14	119.9	C30—C35—H35	120.2
N3—C15—N2	107.0 (2)	N5—C36—N6	107.1 (2)
N3—C15—C16	125.3 (2)	N5—C36—C37	127.9 (2)
N2—C15—C16	127.7 (2)	N6—C36—C37	125.0 (2)
C17—C16—C21	119.9 (2)	C38—C37—C42	119.8 (2)
C17—C16—C15	121.3 (2)	C38—C37—C36	121.0 (2)
C21—C16—C15	118.8 (2)	C42—C37—C36	119.2 (2)
C18—C17—C16	118.0 (2)	C39—C38—C37	117.9 (2)
C18—C17—H17	121.0	C39—C38—H38	121.0
C16—C17—H17	121.0	C37—C38—H38	121.0
C19—C18—C17	123.0 (3)	C38—C39—C40	122.9 (2)
C19—C18—N1	118.8 (2)	C38—C39—N4	118.3 (2)
C17—C18—N1	118.2 (2)	C40—C39—N4	118.8 (2)
C20—C19—C18	118.6 (3)	C41—C40—C39	118.5 (2)
C20—C19—H19	120.7	C41—C40—H40	120.8
C18—C19—H19	120.7	C39—C40—H40	120.8
C19—C20—C21	120.2 (3)	C42—C41—C40	120.1 (3)
C19—C20—H20	119.9	C42—C41—H41	120.0
C21—C20—H20	119.9	C40—C41—H41	120.0
C20—C21—C16	120.2 (3)	C41—C42—C37	120.8 (2)
C20—C21—H21	119.9	C41—C42—H42	119.6
C16—C21—H21	119.9	C37—C42—H42	119.6

### Hydrogen-bond geometry (Å, °)

**Table:**

*D*—H···*A*	*D*—H	H···*A*	*D*···*A*	*D*—H···*A*
N3—H3···Cl1	0.86	2.30	3.066 (2)	148.
N5—H5A···Cl1	0.86	2.20	3.045 (2)	166.
N6—H6···Cl2	0.86	2.31	3.061 (2)	146.
N2—H2···Cl2^i^	0.86	2.21	3.048 (2)	163.

Symmetry codes: (i) *x*, *y*, *z*−1.
